# Resuscitation Attempt and Outcomes in Patients With Asystole Out-of-Hospital Cardiac Arrest

**DOI:** 10.1001/jamanetworkopen.2024.45543

**Published:** 2024-11-18

**Authors:** Junki Ishii, Mitsuaki Nishikimi, Kazuya Kikutani, Shingo Ohki, Kohei Ota, Tatsuhiko Anzai, Kunihiko Takahashi, Masashi Okubo, Shinichiro Ohshimo, Taku Iwami, Nobuaki Shime

**Affiliations:** 1 Department of Emergency and Critical Care Medicine, Graduate School of Biomedical and Health Sciences, Hiroshima University, Hiroshima, Japan; 2 Department of Critical Care Medicine, Shonan Kamakura General Hospital, Kanagawa, Japan; 3 Department of Biostatistics, M&D Data Science Center, Tokyo Medical and Dental University, Tokyo, Japan; 4 Department of Emergency Medicine, University of Pittsburgh School of Medicine, Pittsburgh, Pennsylvania; 5 Department of Preventive Services, School of Public Health, Graduate School of Medicine, Kyoto University, Kyoto, Japan

## Abstract

**Importance:**

Little is known about the epidemiology of out-of-hospital cardiac arrest (OHCA) in patients with asystole in countries where prehospital resuscitation is not withheld or terminated.

**Objective:**

To investigate the secular trends in the patient outcomes and advanced life support (ALS) procedures and evaluate the association of ALS procedures with favorable outcomes among patients with OHCA and asystole.

**Design, Setting, and Participants:**

This cohort study analyzed data from a nationwide prospective OHCA registry in Japan. OHCA occurred from June 1, 2014, to December 31, 2020. Adults with an initial rhythm of asystole and OHCA were included in the analysis, which was conducted between July 29, 2022, and August 24, 2024.

**Exposures:**

Year of OHCA and prehospital ALS procedures (advanced airway management [AAM] and intravenous epinephrine administration).

**Main Outcomes and Measures:**

Trends in prehospital and in-hospital ALS procedures and patient outcomes were described using the Jonckheere-Terpstra trend test for continuous variables and the Cochran-Armitage trend test for categorical variables. The primary outcome was a favorable neurological outcome at 30 days. The secondary outcomes included a favorable neurological outcome at 90 days and survival at 30 and 90 days. Associations between prehospital procedures and outcomes were analyzed using time-dependent propensity score and risk-set matching.

**Results:**

Of 60 349 patients with OHCA, 35 843 (59.4%) presented with asystole (median age, 77 [IQR, 64-85] years; 20 573 [57.4%] men). Among these, 33 674 patients (93.9%) underwent ALS procedures, with 67 (0.2%) achieving a favorable neurological outcome at 30 days. No significant trends in the outcomes were noted, except for a decline in return of spontaneous circulation (424 of 1848 [22.9%] to 1178 of 5892 [20.0%]; *P* = .003). Neither AAM (odds ratio [OR], 1.27 [95% CI, 0.76-2.12]; *P* = .36) nor intravenous epinephrine administration (OR, 0.53 [95% CI, 0.24-1.13]; *P* = .10) was associated with a favorable neurological outcome at 30 days, although both were associated with survival at 30 days (ORs, 1.45 [95% CI, 1.21-1.74] and 1.81 [95% CI, 1.44-2.27], respectively; *P* < .001 for both).

**Conclusions and Relevance:**

In this cohort study of patients with OHCA presenting with asystole, the proportion with a favorable neurological outcome at 30 days was substantially low, and no prehospital ALS procedure was associated with a favorable neurological outcome. These findings suggest that discussions regarding implementation of a termination of resuscitation rule for such patients are warranted.

## Key Points

**Question:**

In countries where prehospital resuscitation efforts are not withheld or terminated, what are the secular trends in outcomes among patients with asystole out-of-hospital cardiac arrest (OHCA), and are prehospital advanced life support procedures (advanced airway management and intravenous epinephrine administration) associated with favorable outcomes?

**Findings:**

In this cohort study of 35 843 adult patients with OHCA and initial asystole in Japan, 67 (0.2%) showed a favorable neurological outcome at 30 days, and there was no significant secular trend. Prehospital procedures were associated with survival but not with a favorable neurological outcome at 30 days.

**Meaning:**

These findings suggest that discussions regarding implementation of a termination-of-resuscitation rule for patients with OHCA and asystole are warranted.

## Teaser

This cohort study describes the secular trends in patient outcomes and advanced life support procedures and evaluates the associations between prehospital procedures and outcomes in patients in Japan with out-of-hospital cardiac arrest and an initial rhythm of asystole.

## Introduction

Out-of-hospital cardiac arrest (OHCA) is a major public health problem across the world,^1^ and the outcomes remain poor despite the recent accumulation of scientific knowledge.^2^ In particular, the outcomes of patients with OHCA and an initial cardiac rhythm of asystole are reported to be poorer compared with those of patients with other initial rhythms, such as shockable rhythms or pulseless electrical activity.^3,4^

A few nationwide reports focusing on the outcomes of patients with OHCA and asystole have been published in the literature.^4,5,6^ However, such reports are limited to those from a few countries where emergency medical services (EMS) personnel can either withhold or terminate prehospital resuscitation efforts in patients with OHCA, and there was no evaluation of the association of prehospital resuscitation efforts with favorable patient outcomes. For example, reports from North America and Australia showed that 58.3% to 75.0% of patients with OHCA and asystole received no resuscitative attempts by EMS personnel or underwent termination of resuscitation (TOR) in the field.^4,5^ The low rate of resuscitative attempts for cardiac arrest with an initial rhythm of asystole could have led to inaccurate evaluation of the disease prognosis and effects of treatment. Considering the improved outcomes of patients with OHCA in general,^7,8,9,10,11^ we considered the possibility that the outcomes of patients with an initial rhythm of asystole could also have improved if they underwent full advanced life support (ALS), including prehospital resuscitative procedures. However, no large studies, to our knowledge, have focused on patients with OHCA and an initial rhythm of asystole to investigate the secular trends in the patient outcomes and evaluate the association of the prehospital ALS procedures with the outcomes in countries where the EMS personnel neither withhold nor terminate resuscitation efforts.

Japan is unique in that EMS personnel are largely prohibited from withholding and terminating resuscitative efforts, so that cardiopulmonary resuscitation (CPR) is uniformly performed in all patients with OHCA, regardless of the initial cardiac rhythm.^12^ We hypothesized that the clinical outcomes in patients with OHCA and an initial rhythm of asystole may be relatively superior in countries where no restriction is imposed on prehospital resuscitation efforts, may improve over time, and that prehospital ALS procedures in these patients might be associated with favorable outcomes. The aim of our study was to describe the secular trends in the patient outcomes and ALS procedures and evaluate the associations between them among patients with OHCA and an initial rhythm of asystole.

## Methods

### Study Design

In this cohort study, we conducted a retrospective analysis of the data of patients registered in the nationwide prospective registry of OHCA maintained by the Japanese Association of Acute Medicine (JAAM-OHCA registry). The methodology of the JAAM-OHCA registry is described in eMethods in Supplement 1. We followed the Strengthening the Reporting of Observational Studies in Epidemiology (STROBE) reporting guideline. The study was performed in accordance with the principles of the Declaration of Helsinki^13^ and with the approval of the Institutional Review Board of Hiroshima University, which waived the need to obtain informed consent from the patients or family so as to ensure participant anonymity as stipulated in the Japanese government guidelines.

For this study, we enrolled patients 18 years or older with OHCA and an initial rhythm of asystole who were registered in the JAAM-OHCA registry between June 1, 2014, and December 31, 2020, and whose prehospital (Utstein Style) data were available. Patients who underwent defibrillation with an automated external defibrillator by a lay person and those without involvement of ALS providers in the prehospital resuscitation were excluded.

### EMS System in Japan

The EMS system in Japan has been described in a previous report.^14^ The EMS personnel are not administratively permitted to withhold or terminate prehospital resuscitation efforts in Japan,^12^ except in a few situations including cases in which death is obvious.^15^ A brief summary of the system is shown in eMethods in Supplement 1.

### Data Collection, Definitions, Exposures, and Outcomes

A detailed explanation of data collection, definitions, and exposures is found in eMethods in Supplement 1. The main exposure variables were year of OHCA and prehospital ALS procedures (advanced airway management [AAM] and intravenous [IV] epinephrine administration). The primary outcome was a favorable neurological outcome, defined as a Cerebral Performance Category (CPC) score of 1 or 2,^16^ at 30 days. The secondary outcomes were favorable neurological outcome at 90 days, survival at 30 and 90 days, and return of spontaneous circulation (ROSC) at any time. We also set a CPC score of 3 or less at 30 days, considered by some as a potential indicator of a favorable neurological outcome based on the long-term functional recovery of some of these patients, as another secondary outcome.^17^

### Statistical Analysis

Analyses were conducted between July 29, 2022, and August 24, 2024. Secular trends of the patient characteristics, performance rate of the prehospital and the in-hospital ALS procedures, and the patient outcomes were analyzed by the Jonckheere-Terpstra trend test for continuous variables and the Cochran-Armitage trend test for categorical variables. The analyses were also conducted in 3 subgroups: patients older than 80 years, patients without ROSC at hospital arrival, and patients who were candidates for TOR according to the ALS-TOR rule.^18^ The ALS-TOR rule and TOR in the hospital setting are described in the eMethods in Supplement 1. The ALS-TOR rule has 91.3% to 100% specificity and 98.8% to 100% positive predictive values for mortality.^19,20^ In addition, subgroup analysis for those who showed ROSC at any time was performed.

The associations between performance of prehospital ALS procedures (AAM and IV epinephrine administration) and the patient outcomes were assessed using time-dependent propensity score and risk-set matching analysis.^21^ The propensity score indicating the time-varying probability of receiving the prehospital procedures was calculated by a competing risk time-to-event analysis using the Fine-Gray regression model.^22,23,24,25,26^ Subsequently, we performed 1:1 risk-set matching with replacement for each of the prehospital procedures using the calculated time-dependent propensity score.^27,28^ We fitted a conditional logistic model with matched pairs to calculate the odds ratio (OR) of a favorable neurological outcome and survival with the 95% CI.^29^ The statistical methods are described in detail in the eMethods in Supplement 1.

All reported *P* values were 2-sided, and the statistical significance was set at *P* < .05. All analyses were performed using the R software, version 4.3.1 (R Project for Statistical Computing) and JMP Pro 16 software (SAS Institute).

## Results

**Figure 1.**
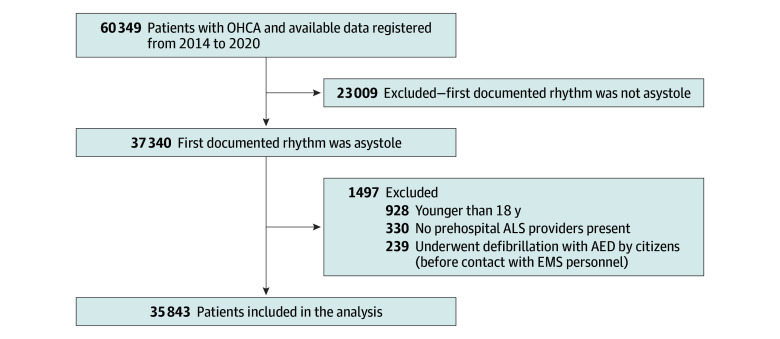
Study Flow Diagram AED indicates automated external defibrillator; ALS, advanced life support; EMS, emergency medical service; and OHCA, out-of-hospital cardiac arrest.

A flowchart of patient enrollment into this study is shown in Figure 1. Among 60 349 patients with OHCA, 23 009 were excluded because they were documented to show an initial nonasystole rhythm. Among the remaining 37 340 patients, 1497 were excluded because they underwent defibrillation with an automated external defibrillator by citizens before the arrival of EMS personnel (n = 239), they were younger than 18 years (n = 928), or no prehospital ALS providers were present at the scene (n = 330).

Among the 35 843 patients included in the analysis (median age, 77 [IQR, 64-85] years; 20 573 [57.4%] men and 15 270 [42.6%] women), 33 674 (93.9%) received some ALS procedures. The baseline characteristics of the patients in each year are summarized in Table 1. The proportion of patients with a presumed cardiac cause increased significantly during the study period (1042 of 1848 [56.4%] in 2014 to 3737 of 5892 [63.4%] in 2020; *P* < .001). The proportion of patients who received bystander CPR increased significantly (775 of 1848 [41.9%] in 2014 to 2925 of 5892 [49.6%] in 2020; *P* < .001).

**Table 1.** Patient Characteristics

Characteristic	Year of OHCA, No. (%) (N = 35 843)							*P* value
	2014 (n = 1848)	2015 (n = 4984)	2016 (n = 5513)	2017 (n = 5706)	2018 (n = 5999)	2019 (n = 5901)	2020 (n = 5892)	
Age, median (IQR), y	76 (63-84)	76 (62-85)	77 (65-85)	77 (65-86)	77 (64-85)	77 (66-85)	77 (65-86)	<.001
Sex								
Men	1054 (57.0)	2888 (57.9)	3123 (56.6)	3247 (56.9)	3512 (58.5)	3381 (57.3)	3368 (57.2)	.90
Women	794 (43.0)	2096 (42.1)	2390 (43.4)	2459 (43.1)	2487 (41.5)	2520 (42.7)	2524 (42.8)	
Cause of cardiac arrest								
Cardiac	1042 (56.4)	3001 (60.2)	3356 (60.9)	3638 (63.8)	3719 (62.0)	3737 (63.3)	3737 (63.4)	<.001
Cerebrovascular	53 (2.9)	116 (2.3)	127 (2.3)	136 (2.4)	118 (2.0)	137 (2.3)	116 (2.0)	.05
Respiratory	141 (7.6)	389 (7.8)	427 (7.7)	353 (6.2)	364 (6.1)	352 (6.0)	352 (6.0)	<.001
Malignant tumor	35 (1.9)	84 (1.7)	73 (1.3)	80 (1.4)	115 (1.9)	86 (1.5)	88 (1.5)	.62
External cause	234 (12.7)	519 (10.4)	625 (11.3)	589 (10.3)	646 (10.8)	571 (9.7)	660 (11.2)	.17
Other or unknown^a^	343 (18.6)	875 (17.6)	905 (16.4)	910 (15.9)	1037 (17.3)	1018 (17.3)	939 (15.9)	.08
Witnessed arrest	499 (27.0)	1375 (27.6)	1549 (28.1)	1539 (27.0)	1599 (26.7)	1656 (28.1)	1627 (27.6)	.77
Bystander CPR	775 (41.9)	2265 (45.4)	2638 (47.9)	2894 (50.7)	2946 (49.1)	2907 (49.3)	2925 (49.6)	<.001
Response time, median (IQR), min^b^	8 (7-10)	8 (7-10)	8 (7-10)	8 (7-10)	8 (7-11)	9 (7-11)	9 (7-11)	<.001

Abbreviations: CPR, cardiopulmonary resuscitation; OHCA, out-of-hospital cardiac arrest.

^a^ Other causes include intoxication, drowning, traffic accident, hypothermia, and anaphylaxis.

^b^ Indicates time from call to contact with the patient.

**Figure 2.**
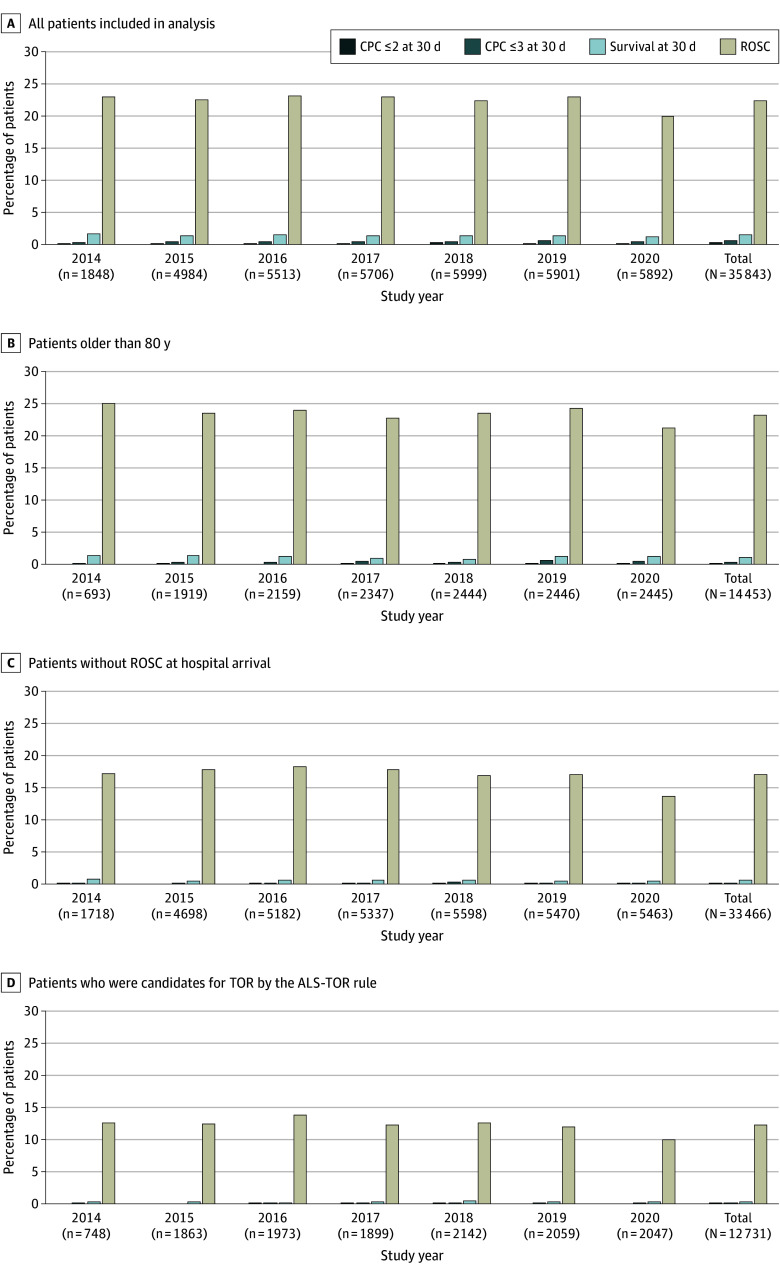
Secular Trends in the Patient Outcomes at 30 Days A Cerebral Performance Category (CPC) score of 2 or less indicates a favorable neurological outcome; a CPC score of 3 or less, a favorable indicator of neurological outcome. ALS-TOR indicates advanced life support–termination of resuscitation; ROSC, return of spontaneous circulation.

Patient outcomes at 30 days and the secular trends are summarized in Figure 2 and eTable 1 in Supplement 1. Among the 35 843 patients, 67 (0.2%) showed a favorable neurological outcome (CPC score ≤2) at 30 days, 172 (0.5%) showed a favorable indicator of neurological outcome (CPC score ≤3) at 30 days, and 497 (1.4%) survived at 30 days. The proportion of patients showing ROSC decreased significantly during the study period (424 of 1848 [22.9%] in 2014 to 1178 of 5892 [20.0%] in 2020; *P* = .003), while there were no statistically significant secular trends in the other outcomes (favorable neurological outcome [CPC score ≤2] at 30 days: 2 of 1848 [0.1%] in 2014 to 11 of 5892 [0.2%] in 2020; *P* = .69) (Figure 2A). We also evaluated the outcomes in certain subgroups, including patients older than 80 years, patients without ROSC at hospital arrival, and patients who were candidates for TOR according to the ALS-TOR rule. Twelve of 14 453 patients older than 80 years (0.1%) and 23 of 33 466 patients without ROSC at hospital arrival (0.1%) showed a favorable neurological outcome (CPC ≤2) at 30 days (Figure 2B and C). Among the 12 731 of 35 843 patients (35.5%) who were candidates for TOR according to the ALS-TOR rule, 8 (0.1%) showed a favorable neurological outcome (CPC score ≤2) at 30 days (Figure 2D). In the analysis of the outcome data at 90 days (26 558 patients [74.2%] without missing data), similar results were obtained for the neurological outcomes at 90 days, while survival at 90 days significantly decreased over the study period, except in patients older than 80 years and those who were candidates for TOR according to the ALS-TOR rule (eFigure 1 in Supplement 1). Among the 8021 of 35 843 patients (22.4%) who showed ROSC at any time during the resuscitation process, 67 (0.8%) showed a favorable neurological outcome (CPC ≤2) at 30 days and 497 (6.2%) survived at 30 days (eFigure 2 in the Supplement).

**Figure 3.**
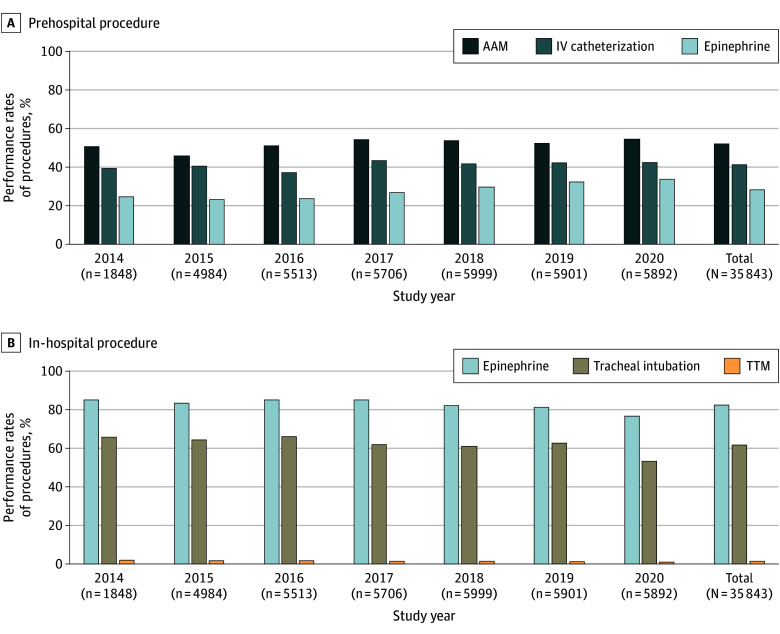
Secular Trends in the Performance of Prehospital and In-Hospital Advanced Life Support (ALS) Procedures in the Analyzed Patients AAM indicates advanced airway management; IV, intravenous; and TTM, targeted temperature management.

Figure 3 and eTable 2 in Supplement 1 illustrate the secular trends in the provision of ALS procedures among the patients included in the analysis. A statistically significant increase was seen in the provision of prehospital ALS procedures (AAM: 938 of 1848 [50.8%] in 2014 to 3216 of 5892 [54.6%] in 2020; IV catheterization: 724 of 1848 [39.2%] in 2014 to 2506 of 5892 [42.5%] in 2020; IV epinephrine administration: 454 of 1848 [24.6%] in 2014 to 1989 of 5892 [33.8%] in 2020; *P* < .001 for all). On the other hand, the provision of in-hospital ALS procedures decreased significantly during the study period (IV epinephrine administration: 1572 of 1848 [85.1%] in 2014 to 4516 of 5892 [76.6%] in 2020; tracheal intubation: 1215 of 1848 [65.7%] in 2014 to 3135 of 5892 [53.2%] in 2020; targeted temperature management: 35 of 1848 [1.9%] in 2014 to 61 of 5892 [1.0%] in 2020; *P* < .001 for all).

To evaluate whether prehospital AAM and epinephrine administration were associated with beneficial outcomes, we performed risk-set matching analyses using the calculated time-dependent propensity score (Table 2). The baseline characteristics of the time-dependent propensity score–matched cohorts for AAM and IV epinephrine administration are shown in eTables 3 and 4, respectively, in Supplement 1. The standardized differences were 0.15 or less for all variables, suggesting a good postmatching balance. For the analyses of AAM and IV epinephrine administration, 18 135 and 9714 patients were matched, respectively. Although both AAM and epinephrine administration were associated with increased survival at 30 days (OR for AAM, 1.45 [95% CI, 1.21-1.74]; OR for IV epinephrine administration, 1.81 [95% CI, 1.44-2.27]; *P* < .001 for both), neither was associated with a favorable neurological outcome (CPC score ≤2) at 30 days (OR for AAM, 1.27 [95% CI, 0.76-2.12; *P* = .36]; OR for IV epinephrine administration, 0.53 [95% CI, 0.24-1.13; *P* = .10]). Similar results were obtained for the outcomes at 90 days, except for a lack of association of AAM with the survival at 90 days (eTable 5 in Supplement 1).

**Table 2.** Association Between Prehospital ALS Procedures and Patient Outcomes at 30 Days

Outcome^a^	AAM			Epinephrine		
	Patients with outcome, No. (%) (n = 18 135)		OR (95% CI)	Patients with outcome, No. (%) (n = 9714)		OR (95% CI)
	Control group	AAM group		Control group	Epinephrine group	
CPC ≤2	26 (0.1)	33 (0.2)	1.27 (0.76-2.12)	19 (0.2)	10 (0.1)	0.53 (0.24-1.13)
CPC ≤3	80 (0.4)	97 (0.5)	1.21 (0.90-1.63)	40 (0.4)	53 (0.5)	1.32 (0.88-2.00)
Survival	202 (1.1)	292 (1.6)	1.45 (1.21-1.74)	116 (1.2)	208 (2.1)	1.81 (1.44-2.27)

Abbreviations: ALS, advanced life support; AAM, advanced airway management; CPC, Cerebral Performance Category; OR, odds ratio.

^a^ A CPC score of 2 or less indicates a favorable neurological outcome; a CPC score of 3 or less, a favorable indicator of neurological outcome.

The time spent on CPR procedures until ROSC is detailed in eTable 6 in the Supplement. A total of 35 434 hours was spent on CPR until ROSC among the analyzed patients, with 529 hours required to achieve 1 favorable neurological outcome (CPC score ≤2) at 30 days. For patients who met the ALS-TOR rule, 12 839 hours (36.2% of total CPR time) were spent on CPR, and 1605 hours were needed for 1 favorable neurological outcome.

## Discussion

From a nationwide prospective observational registry including 35 843 patients with OHCA and an initial rhythm of asystole, this cohort study found that 67 (0.2%) showed a favorable neurological outcome at 30 days, as well as an increasing secular trend in the performance of prehospital ALS procedures. However, neither prehospital AAM nor prehospital epinephrine administration was associated with a favorable neurological outcome at 30 days. A total of 529 hours for CPR procedures were spent to obtain 1 patient with a favorable neurological outcome.

Our study found that the proportion of patients with OHCA and an initial rhythm of asystole who survived and had a favorable neurological outcome was extremely low, with no secular trend toward improvement, even in a modern developed society where EMS personnel are not allowed to withhold or terminate resuscitation efforts. A more pronounced tendency was found in subgroups with poorer outcomes, especially in patients who were candidates for TOR according to the ALS-TOR rule. Among the patients with OHCA and asystole included in the analysis, 35.5% were candidates for TOR, and only 0.1% of these patients showed a favorable neurological outcome (CPC score ≤2) at 30 days, whereas the time for CPR was 12 839 hours (1605 hours per patient with a favorable neurological outcome). Although our results do not entirely preclude the possibility of effective resuscitation even among the subgroups of patients with asystole OHCA and a likelihood of poorer outcomes, we believe that they should provoke discussions about how prehospital TOR might be implemented for patients with asystole OHCA. Of course, clinicians should avoid making prognostic decisions based on the initial rhythm of asystole alone; instead, the prognosis should be based on a combination of several predictive factors besides the initial cardiac rhythm of asystole (eg, witness status, bystander CPR, and cause of the cardiac arrest).^30,31^

Also, our study found that prehospital AAM and epinephrine administration were positively associated with the survival outcome but not with a favorable neurological outcome, which aligned with the results of recent large-scale studies conducted for patients with OHCA and an initial nonshockable rhythm.^22,32^ Despite these results, we did not conclude that prehospital ALS procedures for patients with OHCA and an initial rhythm of asystole were futile because they may not always be futile for individual patients.^33^ Our results should encourage further discussion among regions and cultural spheres. Also, investigation to identify patients in whom a favorable neurological outcome, and not a favorable survival outcome alone, could be expected with prehospital ALS procedures would be of great interest.

The decrease in in-hospital procedures suggests the gradual spread of the concept of pursuing a favorable neurological outcome and better end-of-life care among medical professionals in Japan from the mid-2010s,^15^ with a global growing interest among citizens in concepts such as end-of-life discussions in consideration of an individual’s autonomy, advance care planning, advance directives, and TOR in the context of an aging society.^34,35,36,37^ In contrast, the increased performance of prehospital procedures might be related to the high adherence of EMS personnel to the medical control protocols, which reflects the strict national notification that prehospital ALS procedures should be performed uniformly.

### Limitations

Our study had several limitations. First, we could not fully investigate the effect of the COVID-19 pandemic declared in January 2020 on the secular trends of the patient outcomes and the rate of performance of ALS procedures; we would like to address this in a future work. Second, our primary outcome was the 30-day neurological status. While we also investigated the 90-day neurological status as a secondary outcome, 9285 of 35 843 patients (25.9%) had missing data for this, leading to their exclusion from the analysis. Also, it would be of great interest to investigate the association between ALS procedures and the outcome over a longer term of 90 days or more in the future. Third, there could have been potential confounders not adjusted for when determining the association between prehospital procedures and the patient outcomes that were unavailable from our registry. We could not assess CPR quality, but given the uniform, rigorous training and adherence to resuscitation guidelines by EMS personnel in Japan, suboptimal CPR is unlikely. Fourth, the major etiology of cardiac arrest differs among regions. For example, in Japan, the proportion of patients with a cardiac etiology was high, whereas in North America,^38,39^ it seemed to be lower, with overdose-related OHCA being more widespread.^40^ This difference in the etiology of OHCA could affect the generalizability of our results.

## Conclusions

In this cohort study of patients with OHCA and an initial rhythm of asystole, the proportion of patients with a favorable neurological outcome at 30 days was substantially low (0.2%). Neither prehospital AAM nor epinephrine administration was associated with a favorable neurological outcome at 30 days, although the proportion of patients in whom these procedures were performed increased. These findings suggest that discussions regarding implementation of a TOR rule for such patients are warranted.

## Notes

Supplement 1. **eMethods.** Setting, Data Collection, Definition, Exposures, and Risk-Set Matching Analysis **eTable 1.** Secular Trends in the Patient Outcomes at 30 Days **eFigure 1.** Secular Trends in the Patient Outcomes at 90 Days **eFigure 2.** Secular Trends in the Outcomes Among Patients With ROSC at Any Time **eTable 2.** Secular Trends in the Performance of Prehospital and In-Hospital Advanced Life Support (ALS) Procedures in the Analyzed Patients **eTable 3.** Characteristics of Patients With OHCA and an Initial Rhythm of Asystole Who Received Prehospital AAM and Were at Risk of Receiving AAM in the Time-Dependent Propensity Score-Matched Cohort **eTable 4.** Characteristics of Patients With OHCA and an Initial Rhythm of Asystole Who Received Prehospital Epinephrine Administration and Were at Risk of Receiving Prehospital Epinephrine Administration in the Time-Dependent Propensity Score-Matched Cohort **eTable 5.** Association Between Prehospital ALS Procedures and Patient Outcomes at 90 Days **eTable 6.** Time Spent on CPR Procedures Until ROSC
eReferences.

Supplement 2.
Data Sharing Statement
