# Supplementary material for: Resuscitation Attempt and Outcomes in Patients With Asystole Out-of-Hospital Cardiac Arrest
Source: JAMA Netw Open. 2024 Nov 18;7(11):e2445543. doi: 10.1001/jamanetworkopen.2024.45543 (PMC11574695; doi:10.1001/jamanetworkopen.2024.45543)
Supplement: Supplement 1. — eMethods. Setting, Data Collection, Definition, Exposures, and Risk-Set Matching Analysis eTable 1. Secular Trends in the Patient Outcomes at 30 Days eFigure 1. Secular Trends in the Patient Outcomes at 90 Days eFigure 2. Secular Trends in the Outcomes Among Patients With ROSC at Any Time eTable 2. Secular Trends in the Performance of Prehospital and In-Hospital Advanced Life Support (ALS) Procedures in the Analyzed Patients eTable 3. Characteristics of Patients With OHCA and an Initial Rhythm of Asystole Who Received Prehospital AAM and Were at Risk of Receiving AAM in the Time-Dependent Propensity Score-Matched Cohort eTable 4. Characteristics of Patients With OHCA and an Initial Rhythm of Asystole Who Received Prehospital Epinephrine Administration and Were at Risk of Receiving Prehospital Epinephrine Administration in the Time-Dependent Propensity Score-Matched Cohort eTable 5. Association Between Prehospital ALS Procedures and Patient Outcomes at 90 Days eTable 6. Time Spent on CPR Procedures Until ROSC eReferences. [file jamanetwopen-e2445543-s001.pdf]

Supplemental Online Content

Ishii J, Nishikimi M, Kikutani K, et al. Resuscitation attempt and outcomes among patients with asystole out-of-hospital cardiac arrest. *JAMA Netw Open*. 2024;7(11):e2445543. doi: 10.1001/jamanetworkopen.2024.45543

eMethods. Setting, Data Collection, Definition, Exposures, and Risk-Set Matching Analysis

eTable 1. Secular Trends in the Patient Outcomes at 30 Days

eFigure 1. Secular Trends in the Patient Outcomes at 90 Days

eFigure 2. Secular Trends in the Outcomes Among Patients With ROSC at Any Time

eTable 2. Secular Trends in the Performance of Prehospital and In-Hospital Advanced Life Support (ALS) Procedures in the Analyzed Patients

eTable 3. Characteristics of Patients With OHCA and an Initial Rhythm of Asystole Who Received Prehospital AAM and Were at Risk of Receiving AAM in the Time-Dependent Propensity Score-Matched Cohort

eTable 4. Characteristics of Patients With OHCA and an Initial Rhythm of Asystole Who Received Prehospital Epinephrine Administration and Were at Risk of Receiving Prehospital Epinephrine Administration in the Time-Dependent Propensity Score-Matched Cohort

eTable 5. Association Between Prehospital ALS Procedures and Patient Outcomes at 90 Days

eTable 6. Time Spent on CPR Procedures Until ROSC

eReferences.

© 2024 Ishii J et al. *JAMA Network Open*.

This supplementary material has been provided by the authors to give readers additional information about their work.

© 2024 Ishii J et al. *JAMA Network Open*.

eMethods. Setting, Data Collection, Definition, Exposures, and Risk-Set Matching Analysis

JAAM-OHCA registry

The JAAM-OHCA registry is a nationwide prospective registry of out-of-hospital cardiac arrest (OHCA) patients maintained by the Japanese Association of Acute Medicine (JAAM). The detailed methodology of the JAAM-OHCA registry is described in a previous report.[1] In brief, this registry consists of prehospital data collected using the Utstein-style template[2,3] and in-hospital data, including patient characteristics, resuscitation and intensive care procedures, and outcomes. The neurological status of the survivors was evaluated by the medical staff at each institution 1 month after the event. As quality control, if the data form of the JAAM-OHCA registry was incomplete, the JAAM-OHCA registry committee, composed of specialists in the fields of emergency medicine and epidemiology, returned it to the respective institution requesting that it be completed to the best extent possible, which resulted in no loss of follow-up to determine the outcomes at 30 days among the patients included in this study.

Emergency medical service (EMS) system in Japan

An ambulance carries three emergency providers and at least one emergency life-saving technician who is skilled at intravenous (IV) catheterization, IV epinephrine administration, and advanced airway management (AAM). EMS personnel do not place intraosseous access. Cardiopulmonary resuscitation (CPR) is conducted according to the Japanese guidelines, which are compliant with the international resuscitation guidelines.[4] Termination-of-resuscitation (TOR) can be implemented only when patients satisfy all of the following requirements: patients in a terminal state, patients who have provided advance directive (AD) against resuscitation attempts, patients' family members expressing their wish to withhold CPR, and physicians in charge directing the EMS personnel to terminate CPR in a direct communication. The EMS personnel can withhold CPR only for cases in which death is obvious, such as cases of decapitation, incineration, late postmortem changes (decomposition, mummification, etc) and early postmortem changes (rigor mortis, postmortem lividity, etc).[5]

© 2024 Ishii J et al. *JAMA Network Open*.

Data collection, definition, and exposures

Characteristics of the patients, OHCA event, resuscitative procedures and outcomes were collected from the JAAM-OHCA registry. AAM, IV catheterization, and IV epinephrine administration were defined as the prehospital advanced life support (ALS) procedures. IV epinephrine administration, tracheal intubation, and targeted temperature management (TTM) were defined as in-hospital ALS procedures. The main exposure variables were year of OHCA and prehospital ALS procedures (AAM and IV epinephrine administration). Because of the collinearity between IV catheterization and IV epinephrine administration, we did not include IV catheterization as an exposure variable. In addition, we calculated the time spent on CPR procedures until return of spontaneous circulation (ROSC) was achieved in the prehospital and in-hospital settings as indices of medical resource consumption.

Advanced life support termination-of-resuscitation (ALS-TOR) rule[6] and TOR in the in-hospital setting

Regarding ALS-TOR rule, patients are regarded as candidates for TOR when all the following 5 criteria are met: (1) event not witnessed by the EMS personnel; (2) no automated external defibrillator (AED) used or manual shock applied in the out-of-hospital setting; (3) no ROSC in the out-of-hospital setting; (4) no bystander witness of the arrest; (5) no bystander-administered CPR. As for in-hospital TOR, there are no established criteria in Japan.[4] The Japan Resuscitation Council Resuscitation guidelines 2020 suggest that $EtCO_2$ ≥10 mmHg at 20 minutes after the initial measurement or initiation of resuscitation is a potential predictor of ROSC and survival at hospital discharge.[4] Therefore, physicians often judge whether to terminate or continue resuscitation efforts based on several predictive factors, including the $EtCO_2$, measured at a certain time (such as 20 minutes) after the initiation of resuscitation.

Risk-set matching analysis with time-dependent propensity score (PS)

PS indicating the time-varying probability of receiving the prehospital procedures was calculated by a competing risk time-to-event analysis using the Fine-Gray regression model.[7-11] In the model, time to receiving the procedures was the dependent variable, and arrival of the EMS personnel was set as time 0 because the patients were at-risk of receiving the procedure only after this time-point. We fit two models: one with the dependent variable of time-to-AAM and the other with the dependent variable of time-to-IV

© 2024 Ishii J et al. *JAMA Network Open*.

epinephrine administration. We included age, sex, witness status[7,12] (family, friend, colleague, passers-by, others, or no witness), bystander-initiated CPR, year of occurrence, day of occurrence (weekday or weekend), time of occurrence (daytime or nighttime), external etiology of the cardiac arrest, prehospital involvement of physician and time from the emergency call to arrival of the EMS personnel as the time-independent covariates. In the analysis, we did not include the data of patients who were not in cardiac arrest at the time of arrival of the EMS personnel (but subsequently suffered from cardiac arrest while being treated by the EMS personnel), because patients with a non-asystole rhythm as the first-documented rhythm were excluded from the study. The time-dependent covariates included the remaining prehospital procedures (AAM or IV epinephrine administration) and shock delivery by the EMS personnel.

We performed 1:1 risk-set matching with replacement of each of the prehospital procedures using the calculated time-dependent PS.[13,14] A patient who underwent the prehospital procedures at any given minute after arrival of the EMS personnel was sequentially matched with a patient who was likely to have received prehospital procedures and had a similar PS in the same minute. These at-risk patients could have subsequently undergone the prehospital procedures after the matching or never undergone prehospital procedures, because the matching should be independent of future events.[13,14] At-risk patients could have been matched multiple times as at-risk patients or patients undergoing the prehospital procedures (only if the patients underwent the prehospital procedures) until undergoing the prehospital procedures (matching with replacement).[15] We set the caliper width for the nearest neighbor matching at 0.2 SD of the PS in the logit scale.[16] To assess the performance of the risk-set matching, we calculated a standardized difference for each covariate. A standardized difference of less than 0.25 was regarded as a well-balanced match.[15] We created two PS-matched cohorts: one for AAM and the other for IV epinephrine administration. In each of the matched cohorts, we fitted a conditional logistic model with matched pairs to calculate the odds ratios (ORs) with 95% confidence intervals (CIs).[17] The ORs represented the estimated magnitude of the association of the prehospital procedures with the outcomes as compared with that of those at risk of undergoing prehospital procedures.

© 2024 Ishii J et al. *JAMA Network Open*.

eTable 1. Secular Trends in the Patient Outcomes at 30 days

A All Patients Included in Analysis

	No. (%) of patients (n = 35 843)								
	**2014 (n = 1848)**	**2015 (n = 4984)**	**2016 (n = 5513)**	**2017 (n = 5706)**	**2018 (n = 5999)**	**2019 (n = 5901)**	**2020 (n = 5892)**	***P* value**	**Total (n = 35 843)**
CPC ≤2	2 (0.1)	9 (0.2)	9 (0.2)	12 (0.2)	15 (0.3)	9 (0.2)	11 (0.2)	.69	67 (0.2)
CPC ≤3	5 (0.3)	23 (0.5)	21 (0.4)	27 (0.5)	30 (0.5)	38 (0.6)	28 (0.5)	.11	172 (0.5)
Survival	30 (1.6)	68 (1.4)	87 (1.6)	76 (1.3)	79 (1.3)	84 (1.4)	73 (1.2)	.24	497 (1.4)
ROSC	424 (22.9)	1122 (22.5)	1274 (23.1)	1314 (23.0)	1349 (22.5)	1360 (23.0)	1178 (20.0)	.003	8021 (22.4)

B Patients Older Than 80 years

	No. (%) of patients (n = 14 453)								
	**2014 (n = 693)**	**2015 (n = 1919)**	**2016 (n = 2159)**	**2017 (n = 2347)**	**2018 (n = 2444)**	**2019 (n = 2446)**	**2020 (n = 2445)**	***P* value**	**Total (n = 14 453)**
CPC ≤2	0 (0)	3 (0.2)	0 (0)	3 (0.1)	2 (0.1)	1 (0.04)	3 (0.1)	.81	12 (0.1)
CPC ≤3	1 (0.1)	5 (0.3)	5 (0.2)	9 (0.4)	7 (0.3)	13 (0.5)	10 (0.4)	.08	50 (0.3)
Survival	9 (1.3)	25 (1.3)	27 (1.3)	20 (0.9)	19 (0.8)	30 (1.2)	28 (1.1)	.56	158 (1.1)
ROSC	174 (25.1)	452 (23.6)	517 (23.9)	533 (22.7)	576 (23.6)	596 (24.4)	520 (21.3)	.10	3368 (23.3)

(continued)

© 2024 Ishii J et al. *JAMA Network Open*.

eTable 1. (continued)

C Patients Without ROSC at Hospital Arrival

	No. (%) of patients (n = 33 466)								
	**2014 (n = 1718)**	**2015 (n = 4698)**	**2016 (n = 5182)**	**2017 (n = 5337)**	**2018 (n = 5598)**	**2019 (n = 5470)**	**2020 (n = 5463)**	***P* value**	**Total (n = 33 466)**
CPC ≤2	1 (0.1)	0 (0)	4 (0.1)	5 (0.1)	7 (0.1)	4 (0.1)	2 (0.04)	.57	23 (0.1)
CPC ≤3	2 (0.1)	6 (0.1)	7 (0.1)	11 (0.2)	16 (0.3)	9 (0.2)	6 (0.1)	.74	57 (0.2)
Survival	13 (0.8)	24 (0.5)	36 (0.7)	31 (0.6)	34 (0.6)	29 (0.5)	25 (0.5)	.21	192 (0.6)
ROSC	294 (17.1)	836 (17.8)	943 (18.2)	945 (17.7)	948 (16.9)	929 (17.0)	749 (13.7)	<.001	5644 (16.9)

D Patients Who Were Candidates for TOR by The ALS-TOR Rule

	No. (%) of patients (n = 12 731)								
	**2014 (n = 748)**	**2015 (n = 1863)**	**2016 (n = 1973)**	**2017 (n = 1899)**	**2018 (n = 2142)**	**2019 (n = 2059)**	**2020 (n = 2047)**	***P* value**	**Total (n = 12 731)**
CPC ≤2	0 (0)	0 (0)	2 (0.1)	3 (0.2)	3 (0.1)	0 (0)	0 (0)	.73	8 (0.1)
CPC ≤3	1 (0.1)	0 (0)	2 (0.1)	4 (0.2)	5 (0.2)	1 (0.04)	1 (0.04)	.99	14 (0.1)
Survival	2 (0.3)	5 (0.3)	5 (0.3)	6 (0.3)	9 (0.4)	6 (0.3)	7 (0.3)	.55	40 (0.3)
ROSC	94 (12.6)	231 (12.4)	271 (13.7)	232 (12.2)	269 (12.6)	245 (11.9)	205 (10.0)	.007	1547 (12.2)

Abbreviations: CPC, Cerebral Performance Category; ROSC, return of spontaneous circulation; TOR, termination of resuscitation; ALS-TOR, advanced life support-termination of resuscitation.

© 2024 Ishii J et al. *JAMA Network Open*.

eFigure 1. Secular trends in the patient outcomes at 90 days

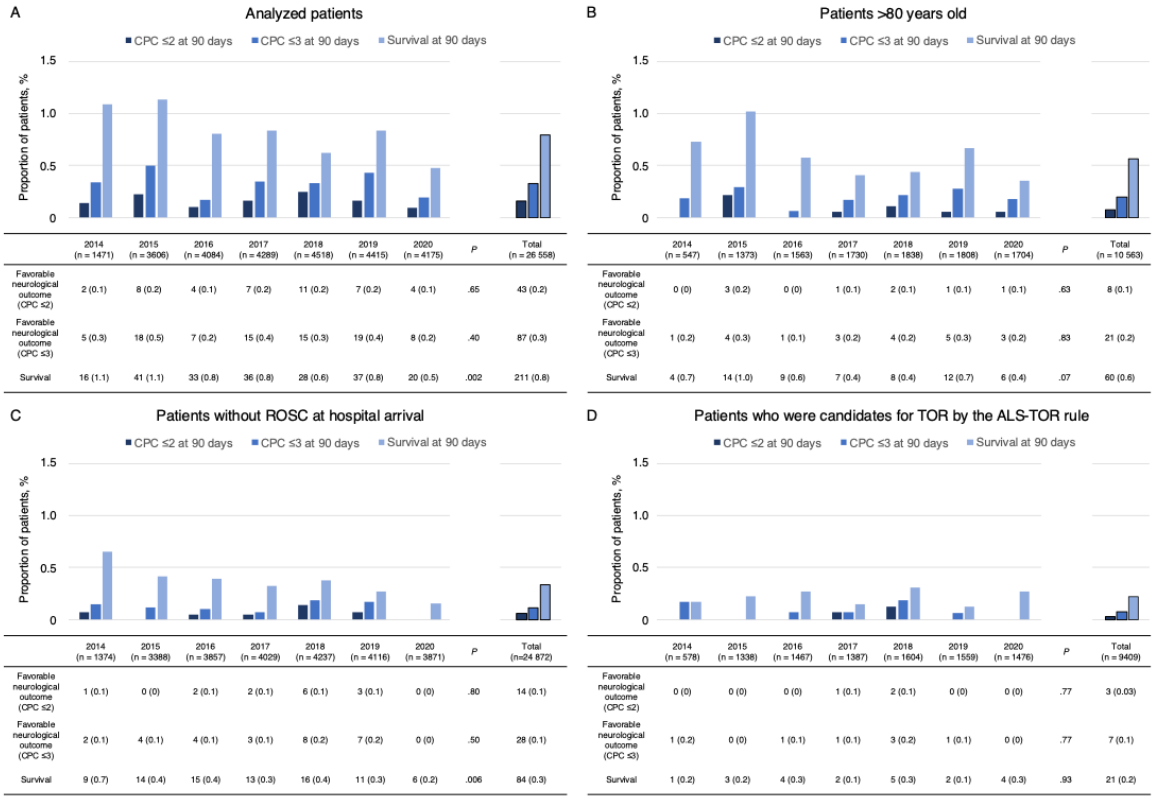

A. Analyzed patients

	2014 (n = 1471)	2015 (n = 3606)	2016 (n = 4084)	2017 (n = 4289)	2018 (n = 4518)	2019 (n = 4415)	2020 (n = 4175)	*P*	Total (n = 26 558)
Favorable neurological outcome (CPC ≤2)	2 (0.1)	8 (0.2)	4 (0.1)	7 (0.2)	11 (0.2)	7 (0.2)	4 (0.1)	.65	43 (0.2)
Favorable neurological outcome (CPC ≤3)	5 (0.3)	18 (0.5)	7 (0.2)	15 (0.4)	15 (0.3)	19 (0.4)	8 (0.2)	.40	87 (0.3)
Survival	16 (1.1)	41 (1.1)	33 (0.8)	36 (0.8)	28 (0.6)	37 (0.8)	20 (0.5)	.002	211 (0.8)

B. Patients >80 years old

	2014 (n = 547)	2015 (n = 1373)	2016 (n = 1563)	2017 (n = 1730)	2018 (n = 1838)	2019 (n = 1808)	2020 (n = 1704)	*P*	Total (n = 10 563)
Favorable neurological outcome (CPC ≤2)	0 (0)	3 (0.2)	0 (0)	1 (0.1)	2 (0.1)	1 (0.1)	1 (0.1)	.63	8 (0.1)
Favorable neurological outcome (CPC ≤3)	1 (0.2)	4 (0.3)	1 (0.1)	3 (0.2)	4 (0.2)	5 (0.3)	3 (0.2)	.83	21 (0.2)
Survival	4 (0.7)	14 (1.0)	9 (0.6)	7 (0.4)	8 (0.4)	12 (0.7)	6 (0.4)	.07	60 (0.6)

C. Patients without ROSC at hospital arrival

	2014 (n = 1374)	2015 (n = 3388)	2016 (n = 3857)	2017 (n = 4029)	2018 (n = 4237)	2019 (n = 4116)	2020 (n = 3871)	*P*	Total (n=24 872)
Favorable neurological outcome (CPC ≤2)	1 (0.1)	0 (0)	2 (0.1)	2 (0.1)	6 (0.1)	3 (0.1)	0 (0)	.80	14 (0.1)
Favorable neurological outcome (CPC ≤3)	2 (0.1)	4 (0.1)	4 (0.1)	3 (0.1)	8 (0.2)	7 (0.2)	0 (0)	.50	28 (0.1)
Survival	9 (0.7)	14 (0.4)	15 (0.4)	13 (0.3)	16 (0.4)	11 (0.3)	6 (0.2)	.006	84 (0.3)

D. Patients who were candidates for TOR by the ALS-TOR rule

	2014 (n = 578)	2015 (n = 1338)	2016 (n = 1467)	2017 (n = 1387)	2018 (n = 1604)	2019 (n = 1559)	2020 (n = 1476)	*P*	Total (n = 9409)
Favorable neurological outcome (CPC ≤2)	0 (0)	0 (0)	0 (0)	1 (0.1)	2 (0.1)	0 (0)	0 (0)	.77	3 (0.03)
Favorable neurological outcome (CPC ≤3)	1 (0.2)	0 (0)	1 (0.1)	1 (0.1)	3 (0.2)	1 (0.1)	0 (0)	.77	7 (0.1)
Survival	1 (0.2)	3 (0.2)	4 (0.3)	2 (0.1)	5 (0.3)	2 (0.1)	4 (0.3)	.93	21 (0.2)

eFigure 1. Secular Trends in the Patient Outcomes at 90 days

A Secular trends in the proportion of patients who showed a favorable neurological outcome (CPC ≤2 and CPC ≤3) at 90 days and survived at 90 days among the analyzed patients.

B Secular trends in the patients aged >80 years old.

C Secular trends in the patients without ROSC at hospital arrival.

D Secular trends in the patients who were candidates for TOR by the ALS-TOR rule.

CPC indicates Cerebral Performance Category; ROSC, return of spontaneous circulation; TOR, termination-of-resuscitation; ALS-TOR, advanced life support termination-of-resuscitation.

© 2024 Ishii J et al. *JAMA Network Open*.

eFigure 2. Secular trends in the outcomes among patients with ROSC at any time

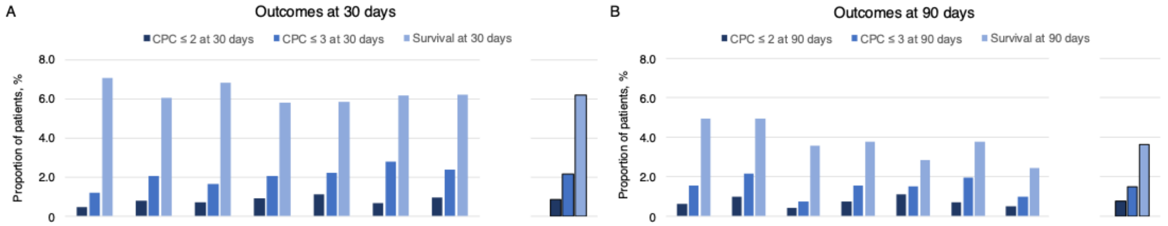

A

	2014 (n = 424)	2015 (n = 1122)	2016 (n = 1274)	2017 (n = 1314)	2018 (n = 1349)	2019 (n = 1360)	2020 (n = 1178)	*P*	Total (n = 8021)
Favorable neurological outcome (CPC ≤2)	2 (0.5)	9 (0.8)	9 (0.7)	12 (0.9)	15 (1.1)	9 (0.7)	11 (0.9)	.52	67 (0.8)
Favorable neurological outcome (CPC ≤3)	5 (1.2)	23 (2.1)	21 (1.6)	27 (2.1)	30 (2.2)	38 (2.8)	28 (2.4)	.04	172 (2.1)
Survival	30 (7.1)	68 (6.1)	87 (6.8)	76 (5.8)	79 (5.9)	84 (6.2)	73 (6.2)	.58	497 (6.2)

B

	2014 (n = 323)	2015 (n = 830)	2016 (n = 929)	2017 (n = 960)	2018 (n = 987)	2019 (n = 979)	2020 (n = 825)	*P*	Total (n = 5833)
Favorable neurological outcome (CPC ≤2)	2 (0.6)	8 (1.0)	4 (0.4)	7 (0.7)	11 (1.1)	7 (0.7)	4 (0.5)	.82	43 (0.7)
Favorable neurological outcome (CPC ≤3)	5 (1.5)	18 (2.2)	7 (0.8)	15 (1.6)	15 (1.5)	19 (1.9)	8 (1.0)	.60	87 (1.5)
Survival	16 (5.0)	41 (4.9)	33 (3.6)	36 (3.8)	28 (2.8)	37 (3.8)	20 (2.4)	.008	211 (3.6)

eFigure 2. Secular Trends in the Outcomes Among Patients With ROSC at Any Time

A Secular trends in the proportion of patients who showed a favorable neurological outcome (CPC ≤2 and CPC ≤3) at 30 days and survived at 30 days among the patients who achieved ROSC at any time during the resuscitation process.

B Secular trends in the proportion of patients who showed a favorable neurological outcome (CPC ≤2 and CPC ≤3) at 90 days and survived at 90 days among the patients who achieved ROSC at any time during the resuscitation process.

ROSC indicates return of spontaneous circulation; CPC, Cerebral Performance Category.

© 2024 Ishii J et al. *JAMA Network Open*.

eTable 2. Secular Trends in the Performance of Prehospital and In-Hospital Advanced Life Support (ALS) Procedures in the Analyzed Patients

A Prehospital Procedure

	**No. (%) of patients (n = 35 843)**								
	**2014 (n = 1848)**	**2015 (n = 4984)**	**2016 (n = 5513)**	**2017 (n = 5706)**	**2018 (n = 5999)**	**2019 (n = 5901)**	**2020 (n = 5892)**	***P* value**	**Total (n = 35 843)**
AAM	938 (50.8)	2279 (45.7)	2815 (51.1)	3090 (54.2)	3224 (53.7)	3090 (52.4)	3216 (54.6)	<.001	18 652 (52.0)
IV catheterization	724 (39.2)	2015 (40.4)	2047 (37.1)	2483 (43.5)	2508 (41.8)	2488 (42.2)	2506 (42.5)	<.001	14 771 (41.2)
Epinephrine	454 (24.6)	1159 (23.3)	1305 (23.7)	1526 (26.7)	1784 (29.7)	1909 (32.4)	1989 (33.8)	<.001	10 126 (28.3)

B In-hospital Procedure

	**No. (%) of patients (n = 35 843)**								
	**2014 (n = 1848)**	**2015 (n = 4984)**	**2016 (n = 5513)**	**2017 (n = 5706)**	**2018 (n = 5999)**	**2019 (n = 5901)**	**2020 (n = 5892)**	***P* value**	**Total (n = 35 843)**
Epinephrine	1572 (85.1)	4153 (83.3)	4687 (85.0)	4854 (85.1)	4939 (82.3)	4798 (81.3)	4516 (76.6)	<.001	29 519 (82.4)
Tracheal intubation	1215 (65.7)	3204 (64.3)	3638 (66.0)	3542 (62.1)	3663 (61.1)	3702 (62.7)	3135 (53.2)	<.001	22 099 (61.7)
TTM	35 (1.9)	88 (1.8)	89 (1.6)	88 (1.5)	85 (1.4)	79 (1.3)	61 (1.0)	<.001	525 (1.5)

Abbreviations: AAM, advanced airway management; IV, intravenous; TTM, targeted temperature management.

© 2024 Ishii J et al. *JAMA Network Open*.

eTable 3. Characteristics of Patients With OHCA and an Initial Rhythm of Asystole Who Received Prehospital AAM and Were at Risk of Receiving AAM in the Time-Dependent Propensity Score-Matched Cohort[a]

	No. (%) of patients		
	**Control group (n = 18 135)**	**AAM group (n = 18 135)**	**Standardized difference**
Age, median (IQR), y	76 (62-85)	78 (67-86)	0.145
Sex			0.010
Male	10 516 (58.0)	10 426 (57.5)	
Female	7619 (42.0)	7709 (42.5)	
External cause of arrest	2188 (12.1)	1391 (7.7)	0.148
Witness category			0.048
Family	2609 (14.4)	3007 (16.6)	
Friend	234 (1.3)	278 (1.5)	
Colleague	124 (0.7)	108 (0.6)	
Passer-by	445 (2.5)	213 (1.2)	
Others	1719 (9.5)	1552 (8.6)	
No witness	13 004 (71.7)	12 977 (71.6)	
Category of bystander CPR			0.052
Chest compression only	7486 (41.3)	7641 (42.1)	
Chest compression with ventilation	709 (3.9)	906 (5.0)	
No bystander CPR	9940 (54.8)	9588 (52.9)	
Year of occurrence			0.035
2014	913 (5.0)	930 (5.1)	
2015	2660 (14.7)	2265 (12.5)	
2016	2776 (15.3)	2781 (15.3)	
2017	2873 (15.8)	3049 (16.8)	
2018	3121 (17.2)	3191 (17.6)	
2019	3022 (16.7)	3053 (16.8)	
2020	2770 (15.3)	2866 (15.8)	

(continued)

© 2024 Ishii J et al. *JAMA Network Open*.

eTable 3. (continued)

	No. (%) of patients		
	Control group (n = 18 135)	AAM group (n = 18 135)	Standardized difference
Day of occurrence			
Weekday	12 052 (66.5)	11 986 (66.1)	0.008
Weekend	6083 (33.5)	6149 (33.9)	
Time of occurrence			
Daytime	7305 (40.3)	7226 (39.8)	0.009
Nighttime	10 830 (59.7)	10 909 (60.2)	
Prehospital involvement of physician	1533 (8.5)	1057 (5.8)	0.102
Response time, median (IQR), min[b]	9 (7-11)	9 (7-10)	0.043

Abbreviations: AAM, advanced airway management; CPR, cardiopulmonary resuscitation.

[a] Continuous variables were expressed as medians (interquartile range [IQR]: 25-75), and categorical variables were expressed as proportions (%).

[b] Time from call to contact with the patient.

© 2024 Ishii J et al. *JAMA Network Open*.

eTable 4. Characteristics of Patients With OHCA and an Initial Rhythm of Asystole Who Received Prehospital Epinephrine Administration and Were at Risk of Receiving Prehospital Epinephrine Administration in the Time-Dependent Propensity Score-Matched Cohort[a]

	No. (%) of patients		
	**Control group (n = 9714)**	**Epinephrine group (n = 9714)**	**Standardized difference**
Age, median (IQR), y	77 (64-85)	78 (67-86)	0.118
Sex			
Male	5575 (57.4)	5772 (59.4)	0.041
Female	4139 (42.6)	3942 (40.6)	
External cause of arrest	1098 (11.3)	714 (7.4)	0.136
Witness category			
Family	1316 (13.5)	2023 (20.8)	0.095
Friend	129 (1.3)	149 (1.5)	
Colleague	74 (0.8)	83 (0.9)	
Passer-by	204 (2.1)	137 (1.4)	
Others	828 (8.5)	1011 (10.4)	
No witness	7163 (73.7)	6311 (65.0)	
Category of bystander CPR			
Chest compression only	4030 (41.5)	4291 (44.2)	0.064
Chest compression with ventilation	431 (4.4)	484 (5.0)	
No bystander CPR	5253 (54.1)	4939 (50.8)	
Year of occurrence			
2014	499 (5.1)	447 (4.6)	0.094
2015	1290 (13.3)	1150 (11.8)	
2016	1483 (15.3)	1274 (13.1)	
2017	1531 (15.8)	1486 (15.3)	
2018	1673 (17.2)	1754 (18.1)	
2019	1691 (17.4)	1868 (19.2)	
2020	1547 (15.9)	1735 (17.9)	

(continued)

© 2024 Ishii J et al. *JAMA Network Open*.

159

© 2024 Ishii J et al. *JAMA Network Open*.

eTable 4. (continued)

	No. (%) of patients		
	Control group (n = 9714)	Epinephrine group (n = 9714)	Standardized difference
Day of occurrence			
Weekday	6467 (66.6)	6479 (66.7)	0.003
Weekend	3247 (33.4)	3235 (33.3)	
Time of occurrence			
Daytime	3859 (39.7)	3994 (41.1)	0.028
Nighttime	5855 (60.3)	5720 (58.9)	
Prehospital involvement of physician	739 (7.6)	687 (7.1)	0.021
Response time, median (IQR), min[b]	8 (7-10)	9 (7-11)	<.001

Abbreviations: CPR, cardiopulmonary resuscitation.

[a] Continuous variables were expressed as medians (interquartile range [IQR]: 25-75), and categorical variables were expressed as proportions (%).

[b] Time from call to contact with the patient.

© 2024 Ishii J et al. *JAMA Network Open*.

eTable 5. Association Between Prehospital ALS Procedures and Patient Outcomes at 90 Days

	AAM			Epinephrine		
	Patients with outcome, No./total No. (%)			Patients with outcome, No./total No. (%)		
**Outcome**	**Control group**	**AAM group**	**OR (95% CI)**	**Control group**	**Epinephrine group**	**OR (95% CI)**
CPC ≤2	17/18 135[a] (0.1)	20/18 135[a] (0.1)	1.67 (0.73-3.81)	14/9714[b] (0.1)	7/9714[b] (0.1)	0.60 (0.22-1.65)
CPC ≤3	41/18 135[a] (0.2)	44/18 135[a] (0.2)	1.29 (0.78-2.11)	22/9714[b] (0.2)	25/9714[b] (0.3)	1.13 (0.57-2.27)
Survival	99/18 135[a] (0.5)	110/18 135[a] (0.6)	1.17 (0.85-1.61)	51/9714[b] (0.5)	80/9714[b] (0.8)	1.54 (1.01-2.36)

[a] There were 9079 missing data for outcome at 90 days in the risk-set matched cohort.

[b] There were 5131 missing data for outcome at 90 days in the risk-set matched cohort.

Abbreviations: ALS, advanced life support; AAM, advanced airway management; OR, odds ratio; CPC, Cerebral Performance Category.

© 2024 Ishii J et al. *JAMA Network Open*.

eTable 6. Time Spent on CPR Procedures Until ROSC

	**Hour (min)**			
	**Analyzed patients (n = 35 843)**		**Patients who were candidates for TOR (n = 12 731)**	
	**Total time**	**Time incurred to obtain one patient with CPC ≤2 at 30 days**	**Total time**	**Time incurred to obtain one patient with CPC ≤2 at 30 days**
Prehospital	20 928[a] (1 255 685)	312[a] (18 742)	7568[d] (454 089)	946[d] (56 761)
In-hospital	14 506[b] (870 348)	217[b] (12 990)	5271[e] (316 266)	659[e] (39 533)
Total	35 434[c] (2 126 033)	529[c] (31 732)	12 839[f] (770 355)	1605[f] (96 294)

[a] There were 5 missing data.

[b] There were 828 missing data.

[c] There was 1 missing data.

[d] There was 1 missing data.

[e] There were 293 missing data.

[f] There was 1 missing data.

Abbreviations: CPR, cardiopulmonary resuscitation; ROSC, return of spontaneous circulation; TOR, termination-of-resuscitation; CPC, Cerebral Performance Category.

© 2024 Ishii J et al. *JAMA Network Open*.

eReferences.

1. Kitamura T, Iwami T, Atsumi T, et al. The profile of Japanese Association for Acute Medicine - out-of-hospital cardiac arrest registry in 2014-2015. *Acute Med Surg*. 2018;5(3):249-258. doi:10.1002/ams2.340
2. Jacobs I, Nadkarni V, Bahr J, et al. Cardiac arrest and cardiopulmonary resuscitation outcome reports: update and simplification of the Utstein templates for resuscitation registries. A statement for healthcare professionals from a task force of the international liaison committee on resuscitation (American Heart Association, European Resuscitation Council, Australian Resuscitation Council, New Zealand Resuscitation Council, Heart and Stroke Foundation of Canada, InterAmerican Heart Foundation, Resuscitation Council of Southern Africa). *Resuscitation*. 2004;63(3):233-249. doi:10.1016/j.resuscitation.2004.09.008
3. Perkins GD, Jacobs IG, Nadkarni VM, et al. Cardiac arrest and cardiopulmonary resuscitation outcome reports: update of the Utstein Resuscitation Registry Templates for Out-of-Hospital Cardiac Arrest: a statement for healthcare professionals from a task force of the International Liaison Committee on Resuscitation (American Heart Association, European Resuscitation Council, Australian and New Zealand Council on Resuscitation, Heart and Stroke Foundation of Canada, InterAmerican Heart Foundation, Resuscitation Council of Southern Africa, Resuscitation Council of Asia); and the American Heart Association Emergency Cardiovascular Care Committee and the Council on Cardiopulmonary, Critical Care, Perioperative and Resuscitation. *Circulation*. 2015;132(13):1286-1300. doi:10.1161/CIR.0000000000000144
4. Japan Resuscitation Council. *JRC Resuscitation Guidelines 2020.* Bunkyo-ku, Tokyo: Igaku Shoin; 2021.

© 2024 Ishii J et al. *JAMA Network Open*.

5. Kajino K, Kitamura T, Iwami T, et al. Current termination of resuscitation (TOR) guidelines predict neurologically favorable outcome in Japan. *Resuscitation*. 2013;84(1):54-59. doi:10.1016/j.resuscitation.2012.05.027

6. Morrison LJ, Verbeek PR, Vermeulen MJ, et al. Derivation and evaluation of a termination of resuscitation clinical prediction rule for advanced life support providers. *Resuscitation*. 2007;74(2):266-275. doi:10.1016/j.resuscitation.2007.01.009

7. Izawa J, Komukai S, Gibo K, et al. Pre-hospital advanced airway management for adults with out-of-hospital cardiac arrest: nationwide cohort study. *BMJ*. 2019;364:l430. doi:10.1136/bmj.l430

8. Matsuyama T, Komukai S, Izawa J, et al. Pre-Hospital Administration of Epinephrine in Pediatric Patients With Out-of-Hospital Cardiac Arrest. *J Am Coll Cardiol*. 2020;75(2):194-204. doi:10.1016/j.jacc.2019.10.052

9. Okubo M, Komukai S, Izawa J, et al. Prehospital advanced airway management for paediatric patients with out-of-hospital cardiac arrest: A nationwide cohort study. *Resuscitation*. 2019;145:175-184. doi:10.1016/j.resuscitation.2019.09.007

10. Matsuyama T, Komukai S, Izawa J, et al. Epinephrine administration for adult out-of-hospital cardiac arrest patients with refractory shockable rhythm: time-dependent propensity score-sequential matching analysis from a nationwide population-based registry. *Eur Heart J Cardiovasc Pharmacother*. 2022;8(3):263-271. doi:10.1093/ehjcvp/pvab013

11. Beyersmann J, Schumacher M. Time-dependent covariates in the proportional subdistribution hazards model for competing risks. *Biostatistics*. 2008;9(4):765-776. doi:10.1093/biostatistics/kxn009

12. Lo CYZ, Fook-Chong S, Shahidah N, et al. Does witness type affect the chance of receiving bystander CPR in out-of-hospital cardiac arrest? *Resuscitation*. 2023;189:109873. doi:10.1016/j.resuscitation.2023.109873

© 2024 Ishii J et al. *JAMA Network Open*.

13. Lu B. Propensity score matching with time-dependent covariates. *Biometrics*. 2005;61(3):721-728. doi:10.1111/j.1541-0420.2005.00356.x

14. Li YP, Propert K.J., Rosenbaum P.R. Balanced Risk Set Matching. *J Am Stat Assoc*. 2001;96(455):870-882

15. Stuart EA. Matching methods for causal inference: A review and a look forward. *Stat Sci*. 2010;25(1):1-21. doi:10.1214/09-STS313

16. Austin PC. An Introduction to Propensity Score Methods for Reducing the Effects of Confounding in Observational Studies. *Multivariate Behav Res*. 2011;46(3):399-424. doi:10.1080/00273171.2011.568786

17. Liu Y, Yo CH, Hu JR, et al. Sepsis increases the risk of in-hospital cardiac arrest: a population-based analysis. *Intern Emerg Med*. 2023. doi:10.1007/s11739-023-03475-6

© 2024 Ishii J et al. *JAMA Network Open*.
