# Supplementary material for: Resuscitation Attempt and Outcomes in Patients With Asystole Out-of-Hospital Cardiac Arrest
Source: JAMA Netw Open. 2024 Nov 18;7(11):e2445543. doi: 10.1001/jamanetworkopen.2024.45543 (PMC11574695; doi:10.1001/jamanetworkopen.2024.45543)
Supplement: Supplement 2. — Data Sharing Statement [file jamanetwopen-e2445543-s002.pdf]

# Data Sharing Statement

Ishii. Resuscitation Attempt and Outcomes in Patients With Asystole Out-of-Hospital Cardiac Arrest. *JAMA Netw Open*. Published November 18, 2024.
doi:10.1001/jamanetworkopen.2024.45543

**Data**
**Data available:** No
